# Can Herbal Medicine Cause Hematoma Enlargement of Hypertensive Intracerebral Hemorrhage within 24 hrs Time Window? A Retrospective Study of 256 Cases from a Single Center in China

**DOI:** 10.1155/2015/868731

**Published:** 2015-02-19

**Authors:** Yafa Xu, Jianwen Guo, Xian Liu, Juehui Li, Jing Wang, Lingbo Hou

**Affiliations:** ^1^No. 1 Neurology Department, Guangdong Province Hospital of TCM, The Second Teaching Hospital of Guangzhou University of Traditional Chinese Medicine, 111 Da'de Road, Yuexiu District, Guangzhou, Guangdong Province 510120, China; ^2^Neurology Department, Qingyuan Traditional Chinese Medicine Hospital, Qingyuan, Guangdong Province 511500, China

## Abstract

A retrospective review was performed of consecutive patients presenting with HICH within 24 hours of ictus presenting between March 2008 and March 2013 who were diagnosed as having HICH by CT scan. Of the 256 patients who matched study inclusion standard, 43 patients hematoma was enlarged (16.8%). The number of the patients who did not take PBC or RBC herbal medicine, took the PBC herbal medicine, and took RBS herbal medicine was 19 (44.2%), 2 (4.7%), and 22 (51.2%) in hematoma enlargement group and 78 (36.6%), 26 (12.2%), and 109 (51.2%) in nonhematoma enlargement group, individually. There was no significant difference between two groups (*P* = 0.671). PBC and RBS herbal medicine did not increase the incidence of hematoma expansion of ICH within 24 hours after onset of symptom.

## 1. Introduction

Hypertensive intracerebral hemorrhage (HICH) is the most devastating form of stroke. Approximately 40% of patients with intracerebral hemorrhage die within 30 days, and the majority of survivors are left with severe disability [1, 2]. Hematoma growth occurs in up to two-third of ICH patients within 24 hours after the onset of symptoms [3]. Furthermore, hemorrhage expansion is an independent determinant of death and disability [4, 5]. Several reasons may be related to the hematoma enlargement in the early stage of HICH, including high blood pressure, “spot” sign of CT scan, sex, age, time window, and anticoagulation drugs [6]. Herbal medicine of promoting blood circulation (PBC) and removing blood stasis (RBS) are widely used in Chinese hospitals to treat HICH; however, whether this herbal medicine can cause hematoma enlargement is undefined until now [7].

In order to evaluate the safety of PBC and RBS herbal drugs, we designed a retrospective study on the hematoma enlargement in HICH patients of 256 cases treated with PBC and RBS herbal medicine within 24-hour time window from the symptom onset in Guangdong Province Hospital of Traditional Chinese Medicine.

## 2. Material and Methods

### 2.1. Materials

A retrospective review was performed of consecutive patients presenting with ICH within 24 hours of ictus presenting between March 2008 and March 2013 who were diagnosed as having hypertensive intracerebral hemorrhage by CT scan. The inclusion criteria also included the following: (1) the patient had hypertensive history; (2) patients got follow-up 24-hour unenhanced CT scan; (3) the patients administrated the herbal medicine within 24 hours from symptom onset of HICH. The exclusion criteria were (1) the time window over 24 hrs from onset to the first CT scan; (2) other reasons causing ICH, such as cerebral tumor, aneurysm, arteriovenous malformation, trauma, anticoagulation drugs, and hematological disorder; (3) lack of second CT scan; (4) lack of other important data of the study. Finally, two hundred and fifty-six cases were reviewed.

### 2.2. Methods

We searched the patients data from the electronic medical record system platform developed by IBM, inc. The searching strategy was “diagnosis=intracerebral hemorrhage” OR “The International Statistical Classification of Diseases and Related Health Problems 10th Revision (ICD-10) [8]” =I61, Intracerebral haemorrhage (Excl.: sequelae of intracerebral haemorrhage) (I69.1) OR I61.0 Intracerebral haemorrhage in hemisphere, subcortical (Deep intracerebral haemorrhage) OR I61.1 Intracerebral haemorrhage in hemisphere, cortical (Cerebral lobe haemorrhage, Superficial intracerebral haemorrhage) OR I61.2 (Intracerebral haemorrhage in hemisphere, unspecified) OR I61.3 (Intracerebral haemorrhage in brain stem) OR I61.4 (Intracerebral haemorrhage in cerebellum) OR I61.5 (Intracerebral haemorrhage, intraventricular) OR I61.6 (Intracerebral haemorrhage, multiple localized) OR I61.8 (Other intracerebral haemorrhage) OR I61.9 (Intracerebral haemorrhage, unspecified), “Admission time=March 2008 to March 2013”, “SEX=BOTH”, “AGE=ALL”.

The patients' raw data were recorded in the well-designed case report forms (CRFs) by two researchers, which contained human demography, medical history, personal history, clinical feather, CT scan, laboratory examination, and herbal medicine treatment. The hematoma volume was measured by ABC/2 Coniglobus formula [9, 10]. Hemorrhage growth was operationally defined as an increase in the volume of intracerebral hemorrhage of >33% as measured by image analysis on the 24-hour CT compared with the baseline CT scan [11].

We defined the herbal medicine as PBC or RBS under the criteria of Chinese Pharmacopoeia of 2010 version. The combined herbal drugs, such as relieving heat and calming liver Yang, decreasing wind and dispersing phlegm, and loosing the bowels, were also under the criteria of Chinese Pharmacopoeia of 2010 version.

Two neuroradiologists analysis on the CT scan data at the work station independently. We divided the patients into hematoma enlargement group and nonhematoma enlargement group. Thus, all the data were analyzed in the statistic software.

### 2.3. Statistical Analysis

Statistical Product and Service Solutions (SPSS Inc.) 19.0 version was used in our study. Firstly, Univariate analysis was used. *χ* ^2^ and nonpaired *t*-tests were used to compare patients with and without hemorrhage growth as to the following variables: age, sex, race, current smoking, prior stroke, diabetes, history of hypertension, blood pressure, location of hemorrhage, volume of ICH on baseline CT, time to first CT scan, baseline platelet count, and baseline prothrombin and partial thromboplastin times. The Wilcoxon rank sum test was used to compare the initial GCS score in patients with and without hemorrhage growth. The Wilcoxon rank sum test was also used to compare patients with and without hemorrhage growth as to the change in the GCS score, hematoma enlargement from baseline to 24 hours. Logistic regression was used to investigate possible multiple risk factors and PBC herbal drugs or RBS herbal drugs for growth in hemorrhage volume from baseline to 24 hours. We also analyzed the possible risk factors and PBC and RBS herbal drugs of 3-month outcome followup (mRS 0-1 as independent outcome, mRS 2–6 as dependent outcome) by logistic regression. All statistical tests were two-tailed, and *P* ≤ 0.05 was considered significant. Data are presented as mean ± SD.

## 3. Results

### 3.1. Patients

Between March 2008 and March 2013, ICH was diagnosed in 901 patients at our hospital. Of these 901 patients, 31 were diagnosed as having bleeding infarction, 43 were diagnosed as having arteriovenous malformation (AVM), 19 were diagnosed as having intracerebral aneurysm, 9 were diagnosed as having cerebral tumor, and 8 were diagnosed as having cerebral trauma. So 791 who were cause by hypertension.

Of the 791 ICH patients, 357 who were admitted after 24 hours of onset were excluded, including 95 patients whose duration was from 24 hrs to 2 ws, 96 patients from 2 ws to 6 ms, and 166 patients more than 6 ms. 70 failed to undergo the second CT because of surgery or death. 108 received emergency surgery within 24 hours after first CT scan. Thus, 256 patients, all of whom underwent the first CT within 24 hours of onset and the second CT within 24 hours after first CT scan, were reviewed in this retrospective study.

**Figure 1:**
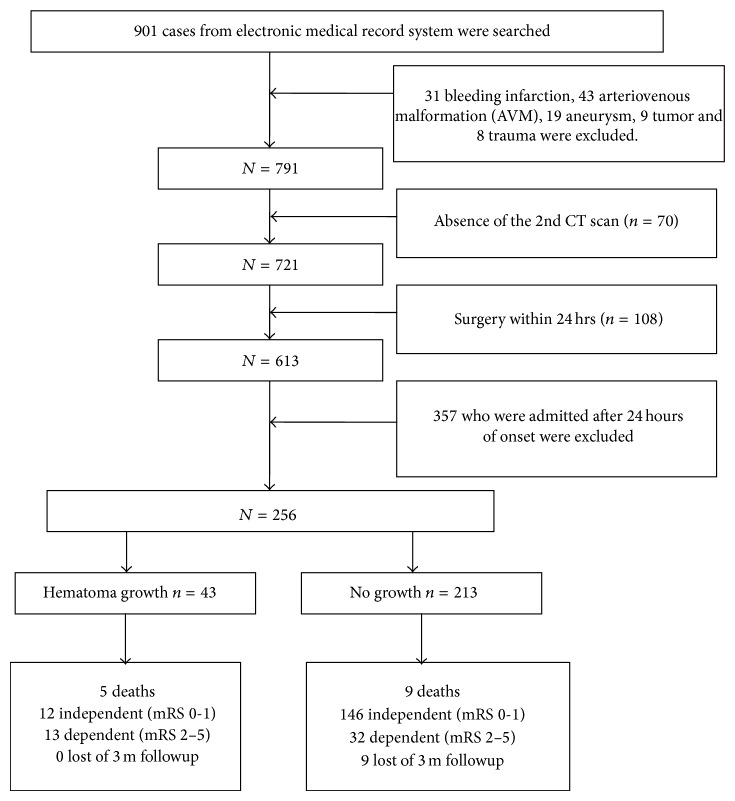
Patients recruited chart.

We also reviewed the patients three months later after entering hospital through telephone or outpatient department visiting. The modified Rankin scale (mRS) was recorded in the followup (see Figure 1).

### 3.2. Hematoma Growth and the Age

Of the 256 patients who matched study inclusion standard, 43 patients hematoma were enlarged (16.8%) within 24 hrs from onset. The mean age was 66 ± 24.00 yrs in hematoma enlargement group and 65.00 ± 22.00 yrs in nonhematoma group; they included 173 male patients and 83 female patients.

### 3.3. PBC and RBS Herbal Medicine Use in Two Groups

**Figure 2:**
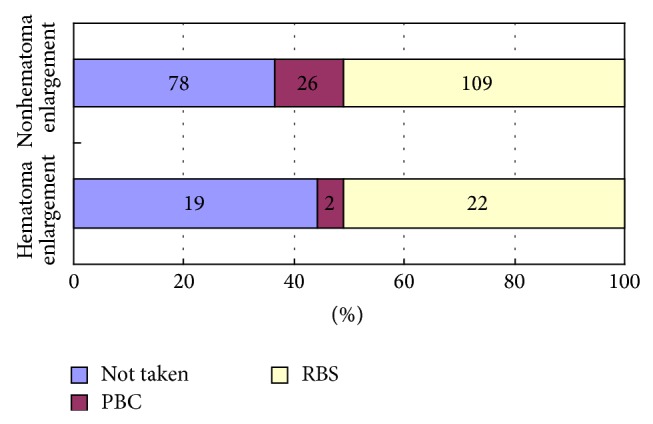
Comparison of PBC and RBS herbal medicine use between two groups.

The number of the patients who did not take the PBC and RBS herbal medicine was 19 (44.2%) in hematoma enlargement group and 78 (36.6%) in nonhematoma enlargement group. The number of the patients who took the PBC and RBS herbal medicine was 24 (55.8%) in hematoma enlargement group and 135 (63.4%) in nonhematoma enlargement group (*P* = 0.390). The number of the patients who took the PBC herbal medicine was 2 (4.7%) in hematoma enlargement group and 26 (12.2%) in nonhematoma enlargement group. The number of the patients who took the RBS herbal medicine were 22 (51.2%) in hematoma enlargement group and 109 (51.2%) in nonhematoma enlargement group. There was no significant difference between two groups (*P* = 0.671). Thus, PBC and RBS herbal medicine could not cause hematoma enlargement of HICH within 24 hrs time window (Figure 2).

### 3.4. Univariate Analysis on the Hematoma Enlargement

We found that patients' sex, baseline GCS, baseline NIHSS, duration from onset to the first CT scan, and aspartate aminotransferase (AST) had significant difference between two groups (*P* < 0.05) (Table 1).

### 3.5. Multivariate Analysis on the Hematoma Enlargement

Patients' sex, baseline Glasgow coma scale, baseline NIHSS, duration from onset to the first CT scan, aspartate aminotransferase (AST), and PBC and RBS herbal medicine use were an independent variable in the multivariate logistic regression analysis and hematoma growth an outcome variable (dependent variable) (Table 2).

There were two independent factors that can cause hematoma growth. The first one was patient's sex (*P* = 0.019). The second one was duration from onset to the first CT scan, 0-1 hr (*P* = 0.046), 1-2 hrs (*P* = 0.041). PBC herbal medicine use (*P* = 0.197) or RBS herbal medicine use (*P* = 0.946) was not independent risk fact. On the other hand, the utilization rate of PBC and RBS herbal medicine was higher in the nonhematoma growth group (63.4%) than in the hematoma growth group (55.8%). The coefficient of regression *β* of RBS herbal medicine use was −1.166, OR = 0.312. The coefficient of regression *β* of PBC herbal medicine use was −0.026, OR = 0.975.

### 3.6. Comparison of Herbal Drugs Combined with PBS and RBC

HICH patients were given herbal drugs formula including several mixed herbal drugs besides PBS and RBS drugs, such as relieving heat and calming liver Yang, decreasing wind and dispersing phlegm, and loosing bowls. We analyse the effect as in Table 3.

The results showed that there was no significant difference between two groups combined with the above three types of herbal drugs (all *P* > 0.05).

### 3.7. Multivariate Analysis on the 3-Month Outcome Followup (mRS)

Patients' sex, baseline Glasgow coma scale, baseline NIHSS, duration from onset to the first CT scan, aspartate aminotransferase (AST), PBC and RBS herbal medicine use, and hematoma growth were an independent variable in the multivariate logistic regression analysis and mRS a dependent variable. We defined mRS 0-1 as independent outcome and mRS 2–6 as dependent outcome.

The results showed that baseline NIHSS and hematoma growth were the independent risk factors of outcome of three-month followup (see Table 4).

There were two independent factors that affect the 3-month outcome. The first one was baseline NIHSS (*P* = 0.000). The second one was hematoma growth (*P* = 0.003). PBC and RBS herbal medicine use was not independent risk factor (*P* = 0.651).

## 4. Discussion

The safety of the herbal medicine administration became more and more critical since aristolochic acids were reported to cause renal injury in 1993 [12, 13].

It is long history that PBC and RBS herbal medicine were used in China to treat HICH. A multicenter, prospective clinical trial showed PBC and RBS herbal medicine can reduce the death and also improve the neurological function [14]. Meta-analysis showed that PBC and RBS herbal medicine seems effective to treat HICH [15]. PBC and RBS were also adopted by textbook and guideline in treating HICH [16].

However, safety data about hematoma enlargement were also reported. Bin and Jian declared that danshen injection and mailuoning injection (one of the PBC and RBS herbal medicine) could induce the uncontrolled bleeding [17]. Leech prevents not only fibrinogen clotting but also other thrombin-catalyzed hemostatic reactions such as the activation of clotting factors V, VIII, and XIII and the thrombin-induced platelet activation [18]. Other researchers advocated that PBC and RBC herbal medicine should be used with few side effects in the clinical application because they added to some other stopping bleeding herbal medicine to make the prescription balance [19]. So it is necessary that we perform this study on the safety of treating HICH with PBC and RBS herbal medicine.

In this study, forty-three (16.8%) of the 256 patients demonstrated enlargement of the hematoma after the first CT scan. The growth rate was near the rate 14.3% (60/419) of Fujii et al.'s report [20]. In this study, 159 patients were administrated PBC and RBS herbal medicine prescription within 24-hour time window, including 24 patients in hematoma enlargement group and 135 patients in no hematoma group, which were not significantly different (*P* > 0.05).

Hematoma enlargement in HICH has significant associations with the duration of time since onset of neurological symptoms, the shape and volume of the bleeding, the initials deep coma degree, the presence of liver dysfunction, and male patients [20, 21]. In this study, the univariate analysis showed that duration, baseline GCS and NIHSS, the liver dysfunction (AST), and male patients were the risk factor of hematoma expansion, while the shape and size of the hematoma were not significant because the sample was too small.

There were only two risk factors that induced hematoma growth according to the multivariate analysis, male patients and duration of onset since ICH symptoms. Hematoma enlargement was the independent risk factor of outcome of three-month followup; the result was similar as Seiji Kazui's study [22]. PBC and RBS herbal drug was not the independent risk factor of hematoma growth of the outcome of three-month followup.

Some herbal drugs have strong PBC and RBS function; for example,* leech* caused rebleeding [21]. Some others have two-way adjustment pharmacological effect; for example,* Radix notoginseng* can not only PBC but also stopping bleeding. In our opinion, the prescription of Chinese medicine to learn is through reasonable compatibility other than a single drug, to eliminate this rebleeding risk.

PBC and RBS herbal medicine was administrated within the time window that was uncertain. A majority (83%) of patients with hematoma enlargement underwent the initial CT scan within 6 hours of onset; Enlargement after 24 hours of onset seems extremely rare [22]. So some neurologists showed their opinion that PBC and RBS herbal medicine should be used after 24 hrs of onset in order to prevent rebleeding risk [23]. Others supported that PBC and RBS herbal medicine should be administrated as sooner as possible [24]. Guo and his colleagues' study did not show deterioration of condition of the AICH patients who were treated with herbal compound within 6 hrs time window from onset [25].

PBC and RBS herbal medicine should be combined with other drugs correctly in the Traditional Chinese Medicine Formula in order to avoid the rebleeding risk, as recorded in the Chinese Pharmacopoeia of 2010 version [26]. In this study, the treatment of acute cerebral hemorrhage frequently used herbal medicine by turns as follows:* Leonurus japonicus* Houtt. (148 cases),* Rhizoma Polygoni Cuspidati* (131 cases), leech (131 cases),* Radix Achyranthis Bidentatae* (83 cases),* Ligusticum chuanxiong* Hort. (21 cases),* Radix Salviae Miltiorrhizae* (18 cases),* Radix et Rhizoma Rhei Palmati* (6 cases),* Cortex moutan* (15 cases), peach seed (13 cases),* Radix Curcumae Wenyujin* (12 cases),* Radix notoginseng* (6 cases),* Carthamus tinctorius* L. (5 cases),* Caulis Spatholobi* (4 cases), and* Rhizoma corydalis* (2 cases). They were used in balance between two groups (*P* > 0.05).

In this retrospective study, 159 patients (24 in hematoma growth group and 135 in no hematoma growth group) were given PBC and RBS herbal medicine prescription, which was also combined with other herbal drugs (showed as Table 4). PBC and RBS drugs reasonable compatibility with other herbs perhaps reduced the risk of hematoma expansion caused by single PBC and RBS herb. This result undoubtedly is instructive for further clinical application.

This is the first retrospective study of the hematoma growth on the early HICH treated with traditional Chinese medicine since now. Though the results showed it seems safe, the retrospective study has many limitations. Firstly, there are too many herbals medication to control the quality of the clinical study. Secondly, a lot of patients were excluded because of important data absent, for example, their second CT scan data. Thirdly, some scales, including NIHSS and GCS, had recall bias from raw medical records.

In order to make up for these limitations, we have designed a prospective, 13 hospitals, randomized, placebo control clinical trial (clinicaltrials.gov: NCT01918722) to confirm if PBC and RBS herbal medicine induces the incidence of hematoma enlargement of AICH patient within the 6 hrs time window from onset. 62 cases have been recruited since February 2014 and all 300 patients will be completed in December 2015.

## Figures and Tables

**Table 1:** The univariate analysis on the hematoma enlargement (%).

Factor	Hematoma enlargement (%) (*n* = 43)	Nonhematoma enlargement (%) (*n* = 213)	Value	*P*
Age (yrs)	66.00 ± 24.00	65.00 ± 22.00	−0.578^△^	0.563
Male	35 (81.4)	138 (64.8)	4.503^⋄^	0.034^*^
Hypertension history	27 (62.80)	139 (65.30)	0.096^⋄^	0.757
DM history	4 (9.30)	25 (11.70)	0.038^●^	0.845
ICH history	4 (9.30)	13 (6.10)	0.591^⋄^	0.442
Alcohol intake				
Yes	11 (25.6)	44 (20.7)		
Stopped	1 (2.3)	10 (4.7)	0.903^⋄^	0.637
No	31 (72.1)	159 (74.6)		
Smoking				
Yes	14 (32.60)	47 (22.10)		
Stopped	3 (7.00)	35 (16.40)	3.814^⋄^	0.149
No	26 (60.50)	131 (61.50)		
Duration from onset to the first CT scan (hrs)				
0~1	6 (14.0)	10 (4.7)		
>1~2	9 (20.90)	20 (9.40)		
>2~4	13 (30.20)	59 (27.70)	9.952^#^	0.002^*^
>4~6	3 (7.00)	26 (12.20)		
>6~24	12 (27.90)	98 (46.00)		
Baseline systolic blood pressure (mmHg)	176.42 ± 31.09	167.82 ± 25.84	−1.920^▲^	0.056
Baseline GCS				
3~7	4 (9.30)	4 (1.90)		
8~13	17 (39.5)	45 (21.1)	12.995^#^	0.000^*^
14~15	22 (51.2)	164 (77.0)		
Baseline NIHSS	10 ± 6	5 ± 7	3.791^△^	0.000^*^
Hematoma location				
Basal ganglion	19 (44.2)	116 (54.4)		
Thalamus	4 (9.3)	37 (17.4)		
Lobar	16 (37.2)	46 (21.6)	6.667^⋄^	0.155
Cerebellar	1 (2.3)	6 (2.8)		
Brain stem	3 (7.0)	8 (3.8)		
Intraventricular hemorrhage	5 (11.6)	28 (13.1)	0.073^⋄^	0.786
Hematoma volume (mL)				
≤15	28 (65.1)	166 (77.9)		
>15~30	9 (20.9)	33 (15.5)	3.290^#^	0.070
>30	6 (14.0)	14 (6.5)		
Irregular hematoma	34 (79.1)	139 (65.3)	3.115^⋄^	0.078
PLT (10^9^/L)	203.00 ± 82.00	209.00 ± 63.50	−0.768^△^	0.443
PT (s)	11.50 ± 1.80	12.00 ± 1.80	−0.270^△^	0.787
APTT (s)	30.00 ± 11.20	30.00 ± 9.25	−0.026^△^	0.979
FIB (g/L)	3.07 ± 0.63	3.09 ± 0.71	−0.466^△^	0.641
ALT (IU/L)	28.00 ± 17.00	21.00 ± 17.00	1.525^△^	0.127
AST (IU/L)	29.00 ± 11.00	24.00 ± 14.00	1.969^△^	0.049^*^
Urea (mmol/L)	5.00 ± 1.44	5.00 ± 1.72	−0.285^△^	0.775
Crea (mmol/L)	80.00 ± 28.00	80.00 ± 23.50	0.388^△^	0.698
PBC and RBS herbal				
Not used	19 (44.2)	78 (36.6)		
PBC	2 (4.7)	26 (12.2)	0.181^#^	0.149
RBS	22 (51.2)	109 (51.2)		
PBC and RBS	24 (55.8)	135 (63.4)	0.870	0.390
Leech	22 (51.2)	109 (51.2)	0.00	1.00
Leonurus	24 (58.1)	124 (58.2)	0.085	0.866
Rhizoma	22 (51.2)	109 (51.2)	0.00	1.00

Note: ^*^ *P* < 0.05, ^▲^ *t*-test, ^△^Mann-Whitney *U* test, ^●^continuous correction chi-square test, ^⋄^Pearson chi-square test, and ^#^Kruskal-Wallis test.

**Table 2:** Multivariate regression analysis on the independent risk factors of hematoma enlargement in 256 patients.

Independent variable	Coefficient of regression	OR	95% CI		*P* value
			Lower	Upper	
Male patient	1.066	2.903	1.189	7.086	0.019^*^
Baseline NIHSS	0.089	1.094	0.993	1.204	0.089
Baseline GCS (14~15)					
Baseline GCS (8~13)	0.970	1.346	0.525	3.451	0.536
Baseline GCS (3~7)	1.054	2.869	0.478	17.238	0.249
Duration (>6~24 h)					
Duration (>4~6 h)	0.106	1.112	0.273	4.524	0.883
Duration (>2~4 h)	0.698	2.009	0.815	4.954	0.130
Duration (>1~2 h)	1.126	3.082	1.046	9.083	0.041^*^
Duration (0~1 h)	1.324	3.759	1.025	13.789	0.046^*^
AST	0.008	1.008	0.997	1.018	0.145
Not used					
RBS	−1.166	0.312	0.053	1.835	0.197
PBC	−0.026	0.975	0.461	2.058	0.946

Note: ∗ means *P* < 0.05.

**Table 3:** Herbal drugs combined with PBS and RBC between two groups (*n*, %).

Combined herbal drugs	PBS and RBC used in hematoma enlargement (*n*, %) (*n* = 24)	PBS and RBC used in nonhematoma enlargement (*n*, %) (*n* = 135)	Value	*P*
Relieving heat and calming liver Yang	21 (87.5)	116 (85.9%)	0.042	0.837
Decreasing wind and dispersing phlegm	14 (58.3%)	104 (77.0%)	3.725	0.054
Loosing bowls	19 (79.2%)	101 (74.8%)	0.208	0.648

**Table 4:** Multivariate regression analysis on the independent risk factors of 3-month outcome in 247 patients.

Independent variable	Coefficient of regression	OR	95% CI		*P* value
			Lower	Upper	
Sex	0.293	1.341	0.630	2.852	0.447
Baseline NIHSS	−0.397	0.672	0.598	0.755	0.000
Baseline GCS	0.211	1.234	0.565	2.697	0.598
Duration from onset	0.022	1.023	0.776	1.347	0.874
AST	−0.006	0.994	0.980	1.009	0.439
PBC and RBS used	0.164	1.178	0.580	2.392	0.651
Hematoma growth	−1.482	0.227	0.085	0.609	0.003
